# Extracellular Phosphate Availability Impacts *Aspergillus terreus* Itaconic Acid Fermentation via Biomass-Specific Product Yield

**DOI:** 10.3390/jof12010014

**Published:** 2025-12-25

**Authors:** Ákos P. Molnár, István Bakondi-Kovács, Vivien Bíró, Alexandra Márton, István S. Kolláth, Erzsébet Fekete, Norbert Ág, Erzsébet Sándor, András Csótó, Béla Kovács, Christian P. Kubicek, Levente Karaffa

**Affiliations:** 1 Department of Biochemical Engineering, Faculty of Science and Technology, University of Debrecen, H-4032 Debrecen, Hungary; molnar.akos@science.unideb.hu (Á.P.M.); kowy63@gmail.com (I.B.-K.); biro.vivien@science.unideb.hu (V.B.); marton.alexandra@science.unideb.hu (A.M.); kollath.istvan91@gmail.com (I.S.K.); ag.norbert@science.unideb.hu (N.Á.); levente.karaffa@science.unideb.hu (L.K.); 2 Institute of Food Science, Faculty of Agricultural and Food Science and Environmental Management, University of Debrecen, H-4032 Debrecen, Hungary; karaffa@agr.unideb.hu (E.S.); kovacsb@agr.unideb.hu (B.K.); 3 Institute of Plant Protection, Faculty of Agricultural and Food Science and Environmental Management, University of Debrecen, H-4032 Debrecen, Hungary; csoto.andras@agr.unideb.hu; 4 Institute of Chemical, Environmental and Bioscience Engineering, TU Wien, 1060 Vienna, Austria; peter.kubicek@tuwien.ac.at

**Keywords:** itaconic acid, *Aspergillus terreus*, fermentation, phosphate ion, D-xylose, D-glucose

## Abstract

Itaconic acid (IA) is an important bio-based platform chemical produced via submerged fermentation by the filamentous Ascomycete *Aspergillus terreus*. In this study, we examined the impact of initial phosphate concentration on IA production from D-glucose and D-xylose in optimized, manganese-limited fermentations. Nine phosphate concentrations ranging from 0.04 to 4 g L^−1^ were tested, and representative low (0.04 g L^−1^), optimal (0.1 g L^−1^), and high (0.8 g L^−1^) conditions were analyzed in detail in controlled, 6 L scale bioreactors. Phosphate availability primarily influenced biomass formation and the biomass-to-product ratio rather than directly affecting IA accumulation. Both lower- and higher-than-optimal phosphate concentrations decreased the volumetric and specific IA yields, while the highest productivity was observed at 0.1 g L^−1^. Expression of the *aoxA* gene, encoding the cyanide-resistant alternative oxidase (AOX), and AOX enzymatic activity were inversely correlated with extracellular phosphate concentration, consistent with a role in redox homeostasis under phosphate-limited conditions. In contrast, total respiration rates and pellet-type morphology remained unaffected. These findings indicate that phosphate acts mainly as a secondary modulator of IA fermentation performance through its influence on biomass formation, whereas other metabolic constraints play a more dominant role in controlling IA overflow in *A. terreus*.

## 1. Introduction

Itaconic acid (IA; methylenesuccinic acid) is a five-carbon, unsaturated, weak diprotic acid (pK_a_ 3.83 and 5.41) with a trifunctional structure (Supplementary Figure S1). Its unique chemical properties are due to the conjugation of one of the two carboxylic acid groups with its methylene group [1]. The thus conjugated double bond of the methylene group will allow polymerization either by addition or condensation [2]. Furthermore, esterification of the two carboxylic groups with different co-monomers offers additional structural and functional versatility, which is exploited by various segments of the polymer industry [3,4,5,6].

On an industrial scale, IA is produced from carbohydrates by submerged fermentation of the filamentous Ascomycete fungus *Aspergillus terreus*, usually in batch mode [1,7,8,9]. Fermentation of IA resembles that of the *A. niger* citric acid bioprocess, which is not surprising given that citric acid is a direct biosynthetic precursor of IA [8,10]. IA overflow requires low pH, high dissolved oxygen, and high initial carbon source concentrations, as well as growth-limiting concentrations of nitrogen and manganese(II) ions, which synergistically increase the yield [8,11,12,13,14].

Dissolved oxygen levels manyfold exceeding those required for maximal biomass formation are needed for high-yield, high-rate IA production [15,16]. Similarly to *A. niger* citric acid fermentation, this phenomenon was proposed to be related to the action of the alternative mitochondrial terminal oxidase [17]. Alternative oxidase (AOX; EC 1.10.3.11) provides an “alternative” for the electron flow opposite to the cytochrome-dependent pathway [18,19]. The branching point is at the level of coenzyme ubiquinone; thus, complexes III and IV of the electron transport chain are bypassed. Consequently, the alternative pathway moves fewer protons across the inner mitochondrial membrane to drive proton motive force, and generates less ATP than by cytochrome-dependent oxidative phosphorylation. We previously identified two putative AOX encoding genes in *A. terreus*, abbreviated as *aoxA* and *aoxB*, *ATEG_05999* and *ATEG_07440*, respectively, and demonstrated that of the two genes, *aoxA* (locus *ATEG_05999*) is solely responsible for the alternative respiration observed under IA producing conditions [15].

Phosphate is an essential macronutient for fungal growth, but its availability is usually restricted during industrial fermentations [9,20]. This is, in general, carried out to limit biomass formation in the presence of high carbon concentrations, but it could as well support specific biochemical mechanisms. Indeed, limiting phosphate ion concentrations in the growth medium can partially alleviate the inhibitory effect of manganese over IA accumulation in *A. terreus* [21,22].

While the IA manufacturing industry predominantly utilizes molasses or corn starch hydrolysates as carbon source, non-food, lignocellulosic plant biomass has become an attractive alternative [23,24,25,26]. Lignocellulose is a complex polymer of hexose and pentose monomers [27,28], with D-xylose being the most abundant pentose [29]. However, relative to hexose-fueled IA fermentations, the biochemical mechanisms that lead to metabolic overflow on D-xylose are less understood.

In this study, we compared fully optimized IA fermentations (i.e., where all cultivation conditions including initial phosphate concentration were optimal for maximal production) with fermentations where initial phosphate concentrations were set higher or lower than the optimal value, resulting in lower yields. Fermentations were performed on D-glucose and D-xylose as respective sole carbon sources. We demonstrate that the initial phosphate concentration primarily affects biomass formation, thereby influencing the biomass-to-product ratio. This ratio (i.e., the biomass-specific IA yield) most accurately reflects the influence of phosphate availability on the overall performance of *A. terreus* IA fermentation. Additionally, the initial phosphate concentration has been shown to be directly proportional to both the activity and expression of the cyanide-resistant alternative oxidase.

## 2. Materials and Methods

### 2.1. Fungal Strain and Cultivation Conditions

*Aspergillus terreus* NRRL 1960 (CBS 116.46; ATCC 10020), a high producer of IA, was maintained on agar plates as described by [3]. The chemically defined minimal medium used throughout the experiments was the same as described earlier [30]. To control the concentration of Mn(II) ions in the growth medium, D-xylose was dissolved in distilled water and passed through a column (440 × 45 mm) of Dowex 50 W-X8 (100/200) (Sigma-Aldrich, St. Louis, MO, USA) cation exchange resin. All components were added to this D-xylose solution from sterile stock solutions. The final Mn(II)-ion concentration was adjusted with MnCl_2_ × 4 H_2_O.

Shake-flask experiments were carried out in 500 mL Erlenmeyer flasks (VWR International Kft., Debrecen, Hungary) containing 100 mL of culture medium. Cultivations were performed at 33 °C on a rotary shaker (Infors AG, Basel, Switzerland) set to 300 rpm, a shaking speed previously demonstrated to ensure adequate aeration for itaconic acid overflow [30]. The starting pH of the medium was adjusted to 3.0 using 3 M HCl.

Bioreactor experiments were conducted in 6 L glass fermenters (Sartorius AG, Göttingen, Germany) with a working volume of 5 L, fitted with two six-blade Rushton disk turbine impellers. Cultivations were performed at 33 °C with an aeration rate of 0.75 vessel volumes per minute (vvm). Prior to inoculation, the medium pH was adjusted to 3.0 using 3 M HCl and was not regulated further during the fermentation. Dissolved oxygen (DO) was maintained at 30% air saturation by adjusting the impeller tip speed as required [15]. DO, temperature, and impeller tip speed were automatically regulated by the bioreactor control system.

To reduce evaporative losses, exhaust gas from the headspace was passed through a reflux condenser connected to an external cooling bath maintained at 4 °C before leaving the reactor. Both shake-flask and bioreactor cultivations were inoculated with 1 × 10^6^ *A. terreus* conidia per milliliter of medium, using a freshly prepared high-density spore suspension in 1:10,000 Tween 20 [30].

All components of the stirring assembly were constructed from high-grade steel, from which Mn^2+^ leaching during sterilization and cultivation was negligible and did not affect the experiments. Nonetheless, Mn^2+^ concentrations were routinely monitored after sterilization and throughout the fermentation. All chemicals were of analytical grade and obtained from Sigma-Aldrich (Budapest, Hungary).

### 2.2. Analytical Methods

Mycelial dry weight (DCW) was quantified from 5 mL culture samples following the method of [31]. Biomass was collected on a pre-weighed glass wool filter, rinsed with cold tap water, and subsequently dried at 80 °C to a constant mass.

D-xylose and itaconic acid (IA) concentrations in the culture supernatants were measured by high-performance liquid chromatography (HPLC; Agilent Technologies, Santa Clara, CA, USA) using a proton-exchange column (Bio-Rad Aminex HPX-87H^+^, Hercules, CA, USA) operated at 55 °C. Isocratic elution was performed with 10 mM H_2_SO_4_, and detection was carried out using a refractive index (RI) detector [32]. Concentrations were calculated as the mean of two independent measurements, which differed by less than 5%. Volumetric IA productivity (g IA L^−1^ h^−1^) was determined from the increase in IA concentration between the initial time point and the time point corresponding to the maximum IA level.

Overall D-xylose consumption rates (g L^−1^ h^−1^) were calculated based on the decrease in residual D-xylose concentration from the start of cultivation until complete depletion of the carbon source. The specific molar IA yield (Y_p/s_) was defined as the molar ratio of IA formed to D-xylose consumed after exhaustion of the substrate. Biomass-specific IA yield (Y_x/s_) was calculated as the ratio of volumetric IA production (g L^−1^) to the DCW determined at the same sampling point. The theoretical carbon balance of the fermentations was estimated using biomass yield coefficients of 0.45 for D-glucose and 0.34 for D-xylose [33], together with specific molar IA yield coefficients of 1.0 for D-glucose and 0.83 for D-xylose.

Manganese(II) ion concentrations in the culture media were determined by inductively coupled plasma quadrupole mass spectrometry (ICP-QMS; Thermo Fisher Scientific, Bremen, Germany) equipped with hexapole collision cell technology (CCT), as described in detail by [11]. Phosphorus concentrations—present exclusively as phosphate in the media—were measured using the same ICP-QMS system. A collision/reaction gas mixture of 7% hydrogen and 93% helium was applied at a flow rate of 6 mL min^−1^. Instrument control was performed using PlasmaLab software (version 2.5.10.319; Thermo Fisher Scientific). Calibration curves were generated from serial dilutions of a mono-elemental phosphorus standard solution (1000 mg L^−1^ P; Scharlab S.L., Sentmenat, Spain), with recoveries ranging between 95% and 100%. Samples were analyzed at *m*/*z* 31 for phosphorus and at *m*/*z* 103 for rhodium, which served as the internal standard at a concentration of 20 μg L^−1^ in all solutions.

Total and cyanide-resistant respiration rates were determined using an oxygraphic electrode (Strathkelvin Instruments Ltd., North Lanarkshire, UK) at 33 °C, in accordance with the manufacturer’s instructions [15]. Following oxygen uptake measurements, the biomass used in these assays was harvested and its DCW determined, enabling calculation of specific oxygen consumption rates. Potassium cyanide was applied at a concentration of 1 mM to selectively inhibit cytochrome c oxidase [34].

Fungal morphology was examined using a Zeiss AxioImager phase-contrast microscope equipped with an AxioCam MRc5 camera (Carl Zeiss, Jena, Germany), as previously described [30].

### 2.3. Total RNA Isolation

Mycelia were harvested by filtration over nylon mesh and washed with sterile dis-tilled water. Excess liquid was removed by squeezing between paper sheets and the bio-mass was quickly frozen in liquid nitrogen. For nucleic acid isolation, frozen biomass was ground to dry powder using a liquid nitrogen-chilled mortar and pestle. Total RNA was isolated with Promega’s SV Total RNA Isolation System (Promega, Fitchburg, WI, USA) [35].

### 2.4. Reverse Transcription Polymerase Chain Reaction (RT-PCR) and cDNA Sequencing

First strand cDNA was synthesized from total RNA template and Oligo(dT) as a pri-mer using the RevertAid First Strand cDNA Synthesis Kit (Thermo Fisher Scientific, Waltham, MA, USA).

### 2.5. Quantitative PCR Analysis

Quantitative PCR (qPCR) was performed on a Rotor-Gene 6500 equipment (Qiagen, Hilden, Germany) with cDNA samples diluted 1:10 in nuclease-free ultrapure water using the Maxima SYBR Green qPCR Master Mix (Thermo Fisher Scientific, MA, USA) [36]. The primers for the analysis are listed in Supplementary Table S1. The suggested cycler program was used for all runs. The log2 fold changes in gene ex-pression at 24, 48 and 72 h between the reference and the high and low phosphate condi-tions were calculated according to the Pfaffl method [37]. The housekeeping gene actA was used as reference gene.

### 2.6. Reproducibility

All presented data are the means of three independent experiments (biological replicates, i.e., new liquid cultures using different spore inocula) and each primary dataset is the mean of two parallel measurements within the same experiment (technical replicates). Data were analyzed and visualized with Sigmaplot software, version 12 (Jandel Scientific, San Rafael, CA, USA), and for all cases standard deviations were determined. Quantitative data (n = 3) were compared using ANOVA with Holm–Sidak Test for pairwise comparisons. The criterion for significance was *p* < 0.05 in all cases.

## 3. Results

### 3.1. Setting Up the Experimental System

To investigate the effects of phosphate on IA accumulation in *A. terreus*, nine different initial KH_2_PO_4_ concentrations taken from the literature (Supplementary Table S2, [3,11,20,21,22,38,39,40,41,42,43]) and spanning between 0.04 and 4 g L^−1^ were tested in a defined growth medium otherwise optimized for IA accumulation in shake-flasks. The concentration of 0.1 g L^−1^—reported optimal for IA production [3] and used as default in our lab [11,14,15,30]—was taken as control. Results showed that both decreasing the phosphate ion concentration to 0.04 g L^−1^ and increasing it to 0.8 g L^−1^ significantly (*p* < 0.05) reduced IA accumulation (measured as volumetric yield) compared to the control condition (Supplementary Table S3). Consequently, the concentrations of 0.04 g L^−1^ and 0.8 g L^−1^—hereafter referred to as “low” and “high” phosphate, respectively—were selected to investigate the effects of non-optimal phosphate levels on IA production under controlled conditions in 6 L scale bioreactors. We emphasize that the phosphate concentrations tested in this study are “optimal” or “higher/lower than the optimal” strictly in regard to IA accumulation only and not to any other aspects of the metabolism or fungal growth in general.

### 3.2. Kinetics of Phosphate Ion Depletion

**Figure 1:**
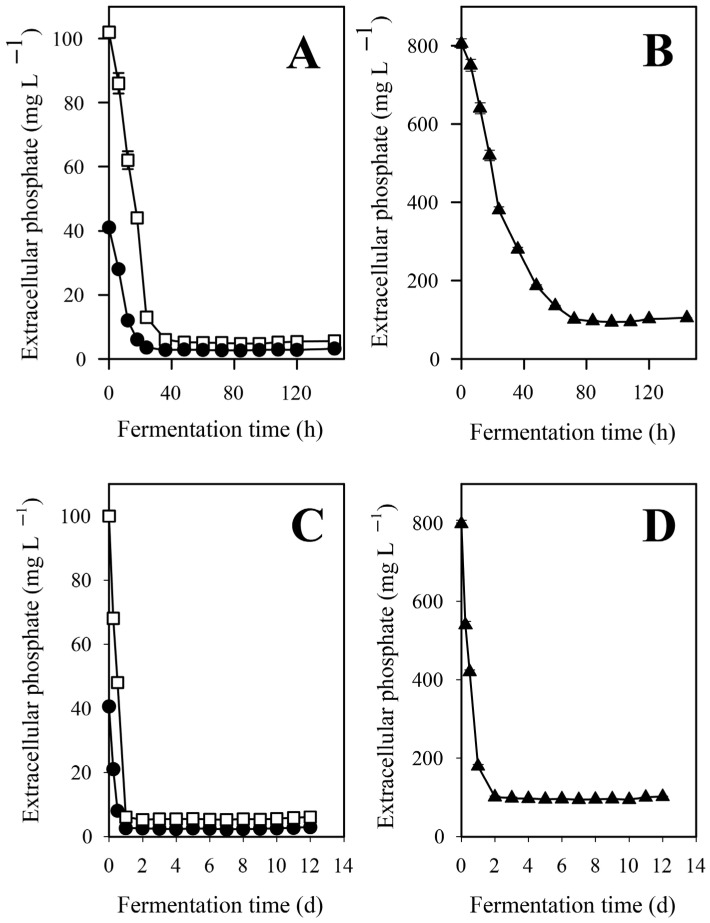
Kinetic profiles of the extracellular phosphate ion concentrations in *Aspergillus terreus* NRRL 1960 cultivations at three different initial phosphate concentrations. Submerged cultures were grown on itaconic acid-producing medium with Mn^2+^ ion deficiency (<3 ppb). Initial concentration of D-xylose was 50 g L^−1^ (Panels **A**,**B**) and that of D-glucose was 120 g L^−1^ (Panels **C**,**D**), respectively, supplementing the medium as sole carbon source. Black circle [●]: 40 mg L^−1^; white square [☐]: 100 mg L^−1^; black triangle [▲]: 800 mg L^−1^ initial phosphate concentration.

The kinetic profile of phosphate ion depletion displayed a biphasic character during conditions optimized for IA accumulation (Figure 1A,B). In the first phase that in the control cultures covered roughly the first two days of the fermentations, some 95% of the 0.1 g L^−1^ initial phosphate was taken up at an average rate of 1.98 ± 0.05 mg L^−1^ h^−1^. This value slightly decreased over the course of the first 48 h. Afterwards, phosphate ion uptake stopped completely, and thus some 5% of the initial phosphate pool remained in the growth medium till the end of the fermentation. Depletion kinetics of both the high (=0.8 g L^−1^) and the low (0.04 g L^−1^) phosphate cultures were qualitatively similar to the control, but the average uptake rate at the first (rapid) phase—that lasted for three and one days, respectively—was 9.72 ± 0.3 g L^−1^ h^−1^ and 1.60 ± 0.03 g L^−1^ h^−1^, respectively. At high phosphate, the biphasic character was less profound, and some uptake could still be detected between the 72–120 fermentation h (Figure 1B). Since growth continued—albeit at lower rate—after uptake was halted, phosphate was likely stored intracellularly to enable biomass formation. This notion was supported by the observation that residual concentrations towards the very end of the fermentations—most notably at the control culture—slightly increased, which was likely caused by the intracellular phosphate released from the disintegrating fungal cells.

### 3.3. High Phosphate Increases Biomass Formation, D-Xylose Consumption but Not Itaconic Acid Accumulation, and Does Not Influence Fungal Morphology

Three parallel IA fermentations (each in triplicate) were performed under identical and optimized cultivation conditions except for the initial phosphate concentration. To assess whether the initial carbon source in the culture medium was utilized solely for biomass and IA formation or if undetected by-products were produced, a carbon balance was established based on theoretical biomass and IA yields. The results indicated that likely no by-products were formed appreciable amounts (Table 1), which was additionally verified by the absence of any by-products in the analysis of the culture supernatant.

**Figure 2:**
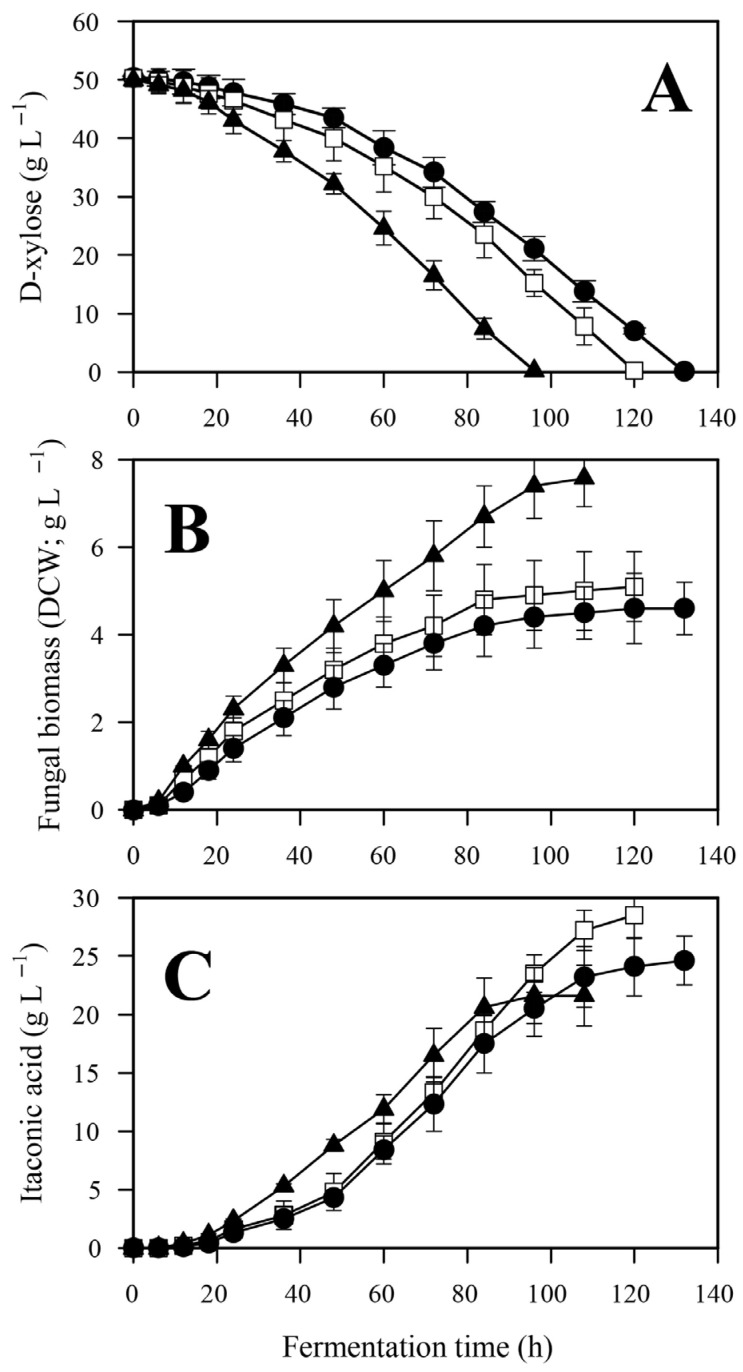
Kinetic profiles of D-xylose (**A**), fungal biomass (DCW, (**B**)), itaconic acid (**C**) in *Aspergillus terreus* NRRL 1960 cultivations at three different initial phosphate concentrations. Submerged cultures were grown on itaconic acid-producing medium with Mn^2+^ ion deficiency (<3 ppb). Initial D-xylose concentration was 50 g L^−1^, supplementing the medium as a sole carbon source. Black circle [●]: 40 mg L^−1^; white square [☐]: 100 mg L^−1^; black triangle [▲]: 800 mg L^−1^ initial phosphate concentration.

Samples were taken every 6–12 h to produce a time-course for biomass (DCW), IA production, and D-xylose consumption (Figure 2); derived parameters were calculated thereof (Table 2).

**Figure 3:**
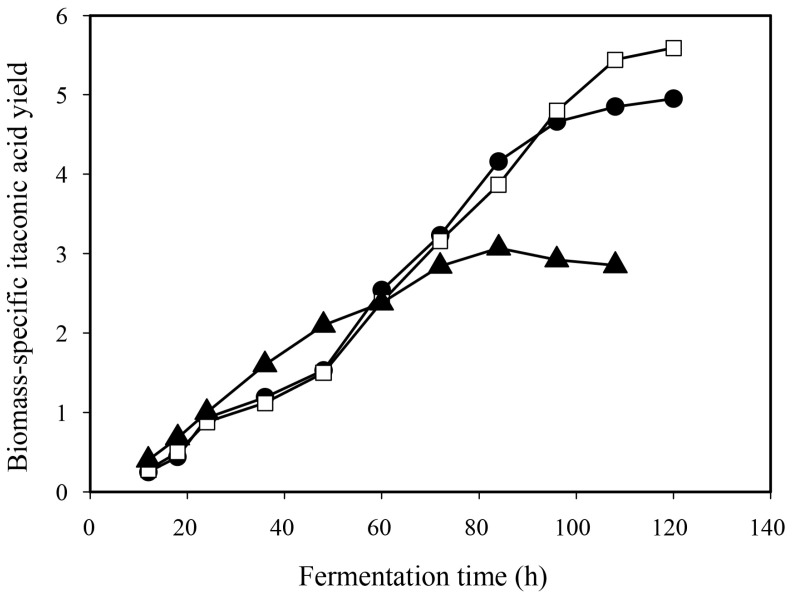
Time-course of the biomass-specific itaconic acid yield (Y_x/s_) of D-xylose-grown *Aspergillus terreus* NRRL 1960 cultures, taken at three different initial phosphate concentrations. Submerged cultures were grown on itaconic acid-producing medium with Mn^2+^ ion deficiency (<3 ppb). Initial D-xylose concentration was 50 g L^−1^, supplementing the medium as a sole carbon source. Black circle [●]: 40 mg L^−1^; white square [☐]: 100 mg L^−1^; black triangle [▲]: 800 mg L^−1^ initial phosphate concentration.

Duration of the IA fermentation—defined by the time needed for the complete depletion of D-xylose—was inversely proportional to the initial phosphate concentration (Figure 2A). Low phosphate cultivation lasted one and a half days longer than the high phosphate cultivation (132 h vs. 94 h), with the duration of the control fermentation falling in between. Consequently, average D-xylose uptake rate was some 20% less at low than at high phosphate conditions, which in turn led to reduced maximal biomass concentration (DCW; Table 2, Figure 2B) as well as reduced biomass formation rate at low phosphate compared to the control and the high phosphate conditions. In contrast, IA accumulation was not observed to be a linear function of the initial phosphate concentration, as both the volumetric and the specific molar yields (Y_x/s_) were highest at the control culture (Table 2, Figure 2C). At low phosphate conditions, both biomass and IA formations significantly (*p* < 0.05) decreased relative to the control, while at high phosphate conditions biomass yield increased at the expense of that of IA yield. The initial phosphate concentration not only influenced the molar yield of the D-xylose—IA bioconversion (Y_p/s_), but also affected product formation rate (g_product_ formed L^−1^ h^−1^). Taken together, these data indicated that the biomass-to-product ratio (i.e., the biomass-specific IA yield; Figure 3) reflects most accurately the influence of the initial phosphate concentration on the performance of *A. terreus* IA fermentation.

**Figure 4:**
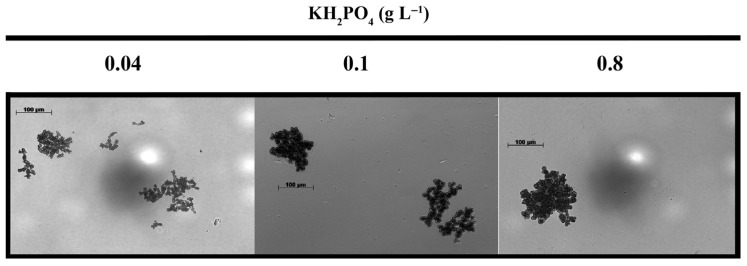
Microscopic images of submerged *Aspergillus terreus* NRRL 1960 cultivations at three different initial phosphate concentrations. Mycelia grown on itaconic acid-producing medium containing initially 50 g L^−1^ D-xylose as a sole carbon source, with Mn^2+^ ion deficiency (<3 ppb). Samples were taken in the early exponential phase (24 h) for analyzing hyphal and pellet diameters. For further details, see Section 3.

The impact of manganese limitation on fungal morphology in IA fermentation is well-documented and shares similarities with *A. niger* citric acid fermentations [8,11]. Under manganese-deficient conditions—as applied throughout this study—the expected morphology dominated by small (<0.5 mm diameter), compact pellets and yeast-like cells was observed irrespective of the initial phosphate levels (Figure 4).

### 3.4. Initial Phosphate Concentration Inversely Correlates to the Expression and Activity of the Alternative Oxidase

**Figure 5:**
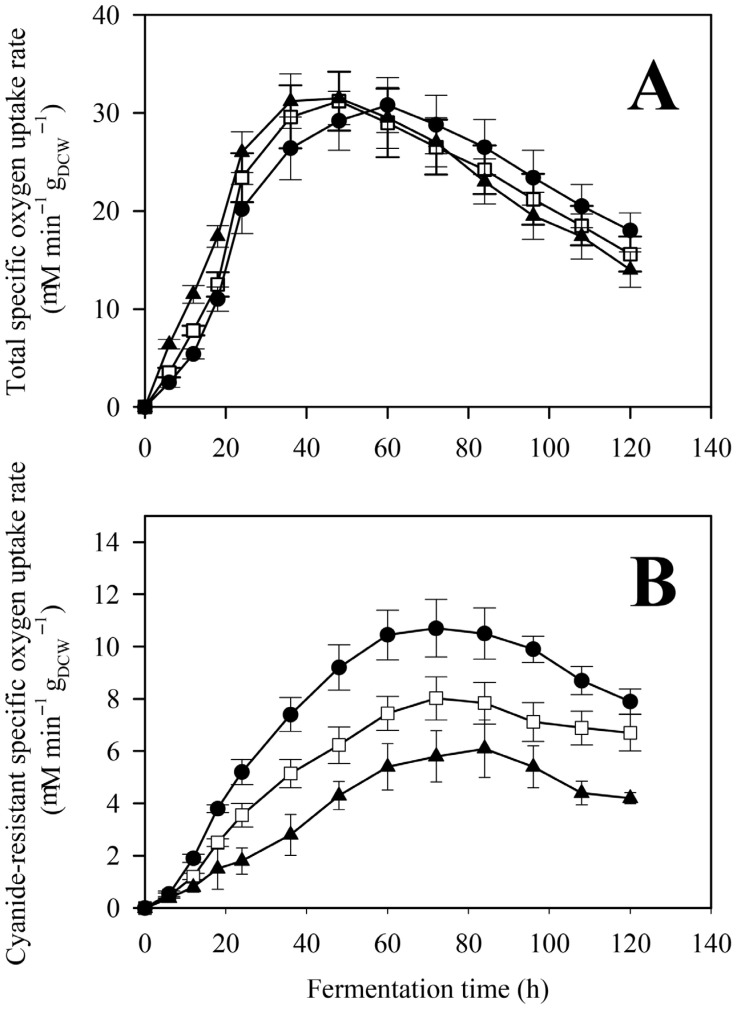
Total (**A**) and cyanide-resistant (**B**) specific respiratory rates in *Aspergillus terreus* NRRL 1960 cultivations at three different initial phosphate concentrations. Submerged cultures were grown on itaconic acid-producing medium using Mn^2+^ ion limitation (<3 ppb). Initial D-xylose concentration was 50 g L^−1^, supplementing the medium as a sole carbon source. Black circle [●]: 40 mg L^−1^; white square [☐]: 100 mg L^−1^; black triangle [▲]: 800 mg L^−1^ initial phosphate concentration.

Overall respiratory rates—defined as the total, uninhibited, dry cell weight-specified oxygen uptake rate—were statistically identical in all three cultivations, independently of the initial phosphate concentration (Figure 5).

**Figure 6:**
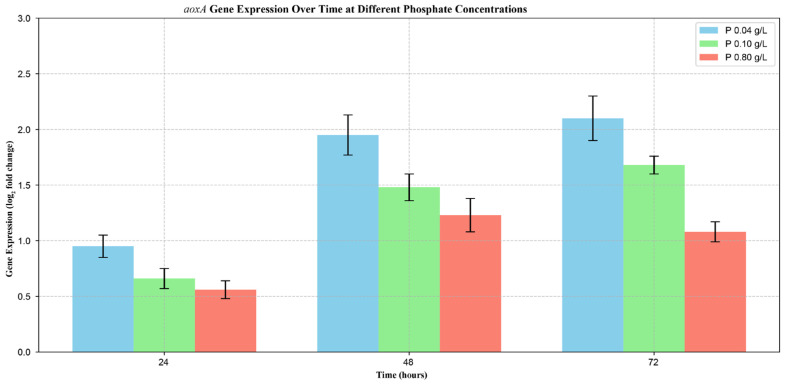
Expression analysis of cyanide-resistant alternative oxidase encoding gene (*aoxA*) over time in *Aspergillus terreus* NRRL 1960 cultivations. Submerged cultures were grown at three different initial phosphate concentrations on itaconic acid-producing medium with Mn^2+^ ion deficiency (<3 ppb). Initial D-xylose concentration was 50 g L^−1^, as a sole carbon source. Samples were taken at 24, 48 and 72 h after the inoculation, respectively. Blue column [■]: 40 mg L^−1^; green column [■]: 100 mg L^−1^; red column [■]: 800 mg L^−1^ initial phosphate concentration.

Respiration rates increased in the first 36 h—coinciding with the rapid growth stage of the fungal cultures—followed by a short plateau then a decline phase (Figure 5A). In contrast, while the kinetic profiles were qualitatively similar, significant (*p* < 0.05) differences were found in the values of the cyanide-resistant oxygen uptake rates (Figure 5B). Alternative respiration rates were inversely proportional to the initial phosphate concentration, with maximal uptake values being almost twice as high in the low as in the high phosphate cultures (10.7 ± 1.1 vs. 6.1 ± 1.1 μM oxygen min^−1^ g_DCW_^−1^), with values from the control fermentation falling in between. These results corroborated the phosphate concentration-dependent expression of *aoxA* (Figure 6), the gene being solely responsible for the cyanide-resistant alternative respiration in *A. terreus* [15]. Expression values were significantly (*p* < 0.05) higher in the low and the control cultures compared to high phosphate conditions.

### 3.5. Effect of the Initial Phosphate Concentration Using D-Glucose as a Sole Carbon Source

Industrial IA fermentations are still predominantly performed using D-glucose-based media [44]. To determine whether phosphate exerts similar effects on the three regulatory levels observed with D-xylose, we repeated the full set of experiments using D-glucose as the sole carbon source. Unlike the D-xylose fermentations, the initial D-glucose concentration was set at 120 g/L, previously identified as optimal [30]. The initial phosphate concentrations were kept identical to those used in the D-xylose experiments.

**Figure 7:**
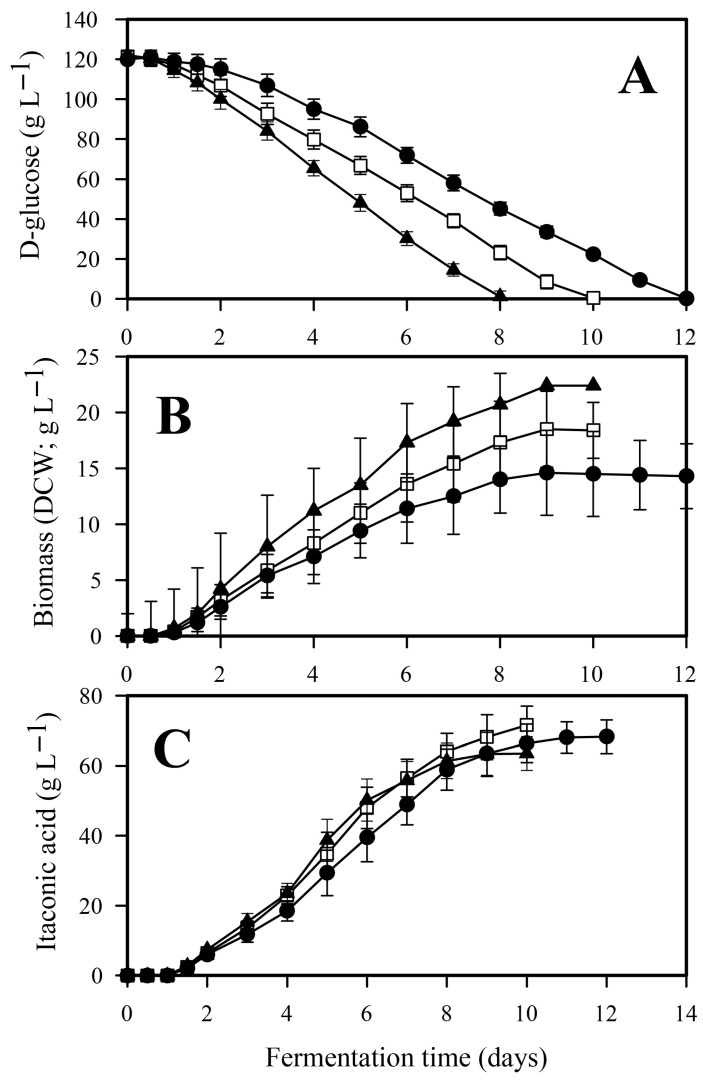
Kinetic profiles of D-glucose (**A**), fungal biomass (DCW, (**B**)) and itaconic acid (**C**) in *Aspergillus terreus* NRRL 1960 cultivations at three different initial phosphate concentrations. Submerged cultures were grown on itaconic acid-producing medium with Mn^2+^ ion deficiency (<3 ppb). Initial D-glucose concentration was 120 g L^−1^, supplementing the medium as a sole carbon source. Black circle [●]: 40 mg L^−1^; white square [☐]: 100 mg L^−1^; black triangle [▲]: 800 mg L^−1^ initial phosphate concentration.

In general, the rates of carbon source consumption, biomass formation, and IA production followed similar qualitative trends to those observed with D-xylose (Figure 7), as did the extracellular phosphate concentration profiles (Figure 1C,D).

**Figure 8:**
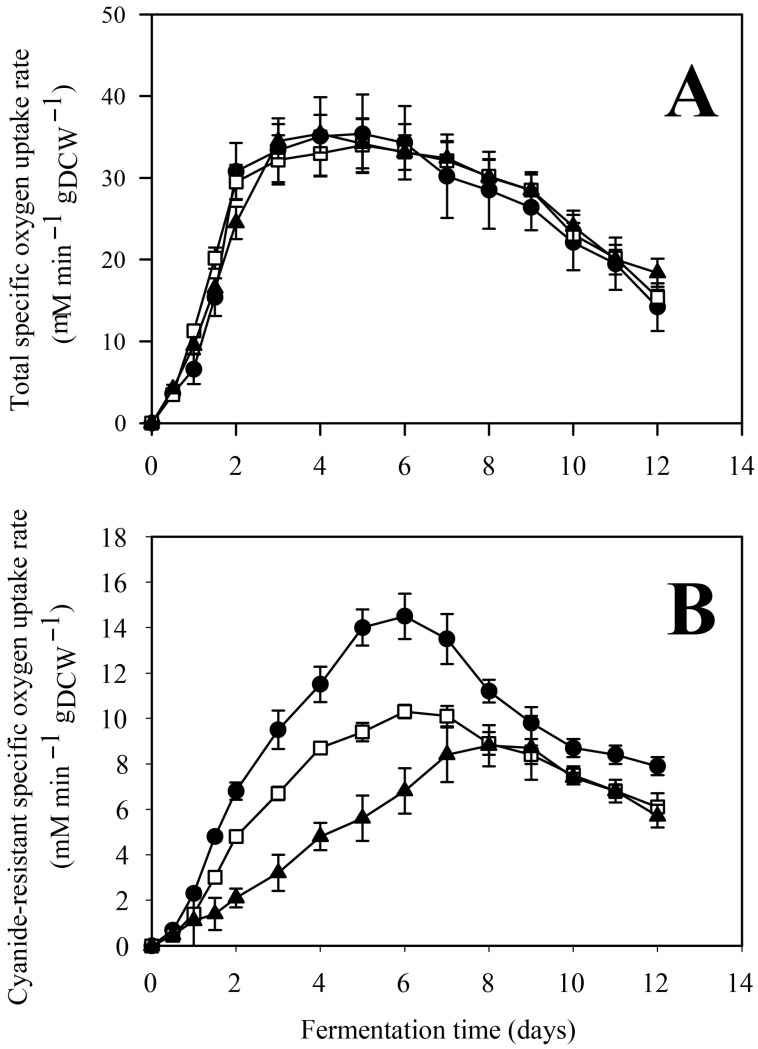
Total (**A**) and cyanide-resistant (**B**) specific respiratory rates in *Aspergillus terreus* NRRL 1960 cultivations at three different initial phosphate concentrations. Submerged culture were grown on itaconic acid-producing medium using Mn^2+^ ion limitation (<3 ppb). Initial D-glucose concentration was 120 g L^−1^, supplementing the medium as a sole carbon source. Black circle [●]: 40 mg L^−1^; white square [☐]: 100 mg L^−1^; black triangle [▲]: 800 mg L^−1^ initial phosphate concentration.

**Figure 9:**
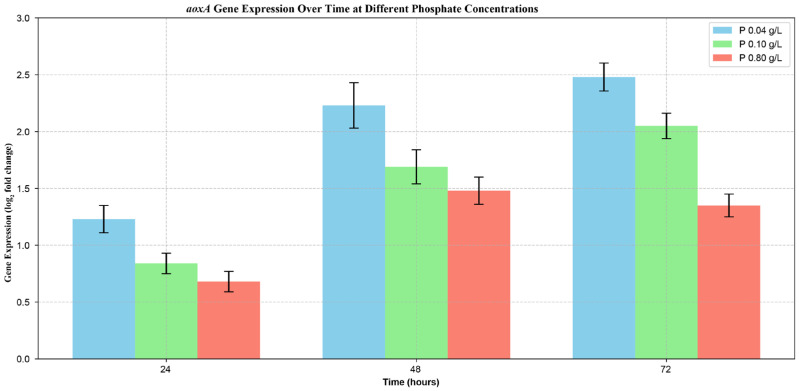
Expression analysis of cyanide-resistant alternative oxidase encoding gene (*aoxA*) over time in *Aspergillus terreus* NRRL 1960 cultivations. Submerged cultures were grown at three different initial phosphate concentrations on itaconic acid-producing medium with Mn^2+^ ion deficiency (<3 ppb). Initial D-glucose concentration was 120 g L^−1^, as a sole carbon source. Samples were taken at 24, 48 and 72 h after the inoculation, respectively. Blue column [■]: 40 mg L^−1^; green column [■]: 100 mg L^−1^; red column [■]: 800 mg L^−1^ initial phosphate concentration.

Both glucose uptake and fungal growth (as indicated by biomass formation) were directly proportional to the initial phosphate concentration. Quantitatively, the specific molar yield of itaconate was higher on D-glucose than on D-xylose, with more pronounced differences between high- and low-phosphate conditions. As observed for D-xylose, biomass accumulation and IA production were consistent with D-glucose consumption (Table 1), with no detectable formation of other byproducts. The activity of the cyanide-resistant alternative oxidase and the expression levels of *aoxA* were also inversely related to the initial phosphate concentration when D-glucose was used as the carbon source (Figure 8 and Figure 9).

As well, D-glucose-grown cultures displayed the typical overflow-associated morphology characterized by small, compact pellets and yeast-like cells throughout the fermentation.

## 4. Discussion

The ‘optimal’ phosphate concentration in fungal cultures is not universal, as it depends on the species and strain, the objective of the culture, and the culture system employed (shake flasks vs. bioreactors, defined vs. complex media). Nevertheless, phosphate concentrations in general fungal culture media are typically reported in the range of 2–10 mM, which supports robust growth and normal metabolic activity [45,46,47,48]. The phosphate concentration of 0.8 g L^−1^ used as the ‘high-phosphate’ condition in this study falls well within the commonly reported optimal range, whereas the lower concentrations of 0.1 and 0.04 g L^−1^ represent phosphate-limited conditions, indicated by reduced biomass formation. Based on this, we tested the hypothesis that phosphate limitation—a well-established determinant of high-yield IA production from D-glucose by *A. terreus* [9,21]—also exerts a regulatory role in D-xylose-based IA overflow. Our results do not support this hypothesis: the main effect of phosphate occurs at a fermentation time when phosphate has already been completely consumed, and the overall differences in IA yields between phosphate limitation and sufficiency are only small. In fact, our data show that phosphate sufficiency leads to higher biomass production and correspondingly less carbon source is available for IA production. The small observed difference implies that metabolic constraints other than phosphate exert a greater impact on IA production, with phosphate influencing the process only marginally through biomass formation. Such a shift is consistent with previous reports on *A. terreus* outlining the effect of different nutrient limitations on IA production [9,21,49]. Although the initial catabolic pathways that lead from D-glucose and D-xylose toward itaconate differ, both pathways converge at the shared PPP/EMPP intermediates fructose-6-phosphate and glyceraldehyde-3-phosphate, after which carbon is processed through similar metabolic routes [50,51]. This convergence may explain why phosphate availability exerts comparable qualitative effects on fermentation performance, regardless of whether D-glucose or D-xylose is supplied as the main substrate.

Our study revealed an inverse proportionality between the initial extracellular phosphate concentration and both aoxA expression and alternative oxidase activity. When phosphate is abundant, ATP synthase operates efficiently, and oxidative phosphorylation is not bottlenecked [52]. The electron transfer chain remains balanced, with less risk of over-reduction in the ubiquinone pool or reactive oxygen species (ROS) buildup [53,54]. Under these conditions, there is no need to activate AOX, so aoxA expression and AOX activity remain low [55,56]. However, under low-phosphate conditions, ATP synthase is impaired because inorganic phosphate is a substrate for ATP production. This causes accumulation of NADH and a highly reduced electron transfer chain, favoring ROS production. AOX induction (aoxA upregulation) provides a bypass that relieves this redox stress by oxidizing ubiquinol without contributing to the proton gradient [57]. By oxidizing excess NADH, AOX helps sustain glycolytic and TCA flux under phosphate-limiting conditions, keeping carbon flowing into IA biosynthesis, which is derepressed when phosphate is scarce. However, extracellular phosphate is strictly inversely related to aoxA expression and activity, whereas its relationship with IA yield is more nuanced: inverse above 0.1 g L^−1^ phosphate but directly proportional below this level. Consequently, AOX cannot be considered a direct mediator of the phosphate effect on IA biosynthesis in *A. terreus*.

Fungal morphology is tightly linked to nutrient availability, including extracellular phosphate concentrations [58,59]. Phosphate limitation—not unlike manganese deficiency applied throughout this study—leads to increased hyphal branching, reduced hyphal diameter, and formation of fragmented mycelial aggregates, with downstream effects on oxygen and nutrient transfer in submerged cultures [60]. These morphologies improve oxygen and nutrient penetration, which can favor the secretion of organic acids [61]. Variations in the initial phosphate concentration had no major effect on the morphology of *A. terreus*—small pellets and yeast-like cells dominated in all three cases. We assume that under conditions favorable for IA overflow, the strong manganese(II) ion limitation was the primary determinant of morphology, and therefore phosphate deviations within the tested range did not play a decisive role.

Recent advances in alternative microbial platforms for itaconic acid production indicate that the phosphate-dependent partitioning of carbon between growth and product formation observed in *A. terreus* may be a general phenomenon. Yeasts (*Yarrowia lipolytica*, *Komagataella phaffii*) and Ustilaginaceae (*U. maydis*, *U. cynodontis*) achieve high titers and productivity through metabolic reprogramming (e.g., overexpression of cis-aconitate decarboxylase, elimination of lipid sinks, transporter engineering) and process optimization using diverse substrates including glucose, waste oils, methanol, and molasses [62,63,64,65,66]. As in *A. terreus*, extracellular phosphate is expected to modulate biomass formation and redox balance, influencing the allocation of carbon between growth and itaconate production. Low phosphate can limit ATP synthesis and increase NADH/NAD^+^ ratios, triggering redox-balancing mechanisms such as alternative respiration that sustain glycolytic and TCA flux and favor carbon overflow toward itaconate [49,52,53,54]. Phosphate sufficiency, conversely, promotes biomass at the expense of product formation. Although phosphate-dependent regulation has not been systematically studied in these alternative hosts, evidence from Ustilago and Yarrowia indicates that nutrient limitation generally favors overflow metabolism and secondary metabolite formation [63,64,65]. These observations suggest that metabolic engineering, process design, and phosphate management should be considered together when optimizing itaconate yields across different microbial platforms.

## Figures and Tables

**Table 1:** Carbon balance in *Aspergillus terreus* NRRL 1960 submerged cultivations at three different initial phosphate concentrations. Mycelia grew in 6 L scale bioreactors in an optimized IA-producing medium initially containing 50 g L^−1^ D-xylose or 120 g L^−1^ D-glucose, as the sole carbon source. Maximum concentrations of biomass (DCW) and itaconate (IA) were empirically determined from three parallel fermentations, and the mean values were used for the theoretical calculations. For clarity, standard deviations are not presented for either the measurements or the calculations. For further information on the calculations (molar itaconate yield, theoretical carbon requirement and carbon balance), see Section 2.

Initial Carbon Source	D-Xylose: 50.4 g L^−1^			D-Glucose: 120.4 g L^−1^		
Initial Phosphate Ion						
Parameters	40 mg L^−1^	100 mg L^−1^	800 mg L^−1^	40 mg L^−1^	100 mg L^−1^	800 mg L^−1^
DCW_max._ (g/L)	4.6	5.1	7.57	14.6	18.5	22.4
Biomass yield (Y_x/s_)	9.1%	10.1%	15.01%	12.1%	15.4%	18.6%
Itaconate_max._ (g/L)	24.6	28.5	21.6	68.3	71.6	63.5
Molar itaconate yield (mol/mol)	0.56	0.65	0.49	0.79	0.82	0.73
Theoretical carbon required for DCW	4.6/0.34 = 13.5	5.1/0.34 = 15	7.57/0.34 = 22.3	14.6/0.45 = 33.4	18.5/0.45 = 41.1	22.4/0.45 = 49.8
Theoretical carbon required for IA	24.6/0.72 = 34.2	28.5/0.72 = 39.6	21.6/0.72 = 30	68.3/0.72 = 94.9	71.6/0.72 = 99.4	63.5/0.72 = 88.2
Overall theoretical carbon requirement (g/L)	13.5 + 34.2 = 47.7	15 + 39.6 = 54.6	22.3 + 30 = 52.3	33.4 + 94.9 = 128.3	41.1 + 99.4 = 140.8	49.8 + 88.2 = 138
Carbon balance, % (theoretical/consumed)	47.7/50.4 = 94.6	54.6/50.4 = 108.3	52.3/50.4 = 103.7	128.3/120.4 = 106.5	140.8/120.4 = 116.9	138/120.4 = 114.6

**Table 2:** Biomass (DCW) and itaconic acid (IA) production, D-xylose uptake as well as derived kinetic parameters of *Aspergillus terreus* NRRL 1960 cultures. Mycelia grew under submerged conditions in 6 L scale bioreactors in an optimized IA-producing medium initially containing 50 g L^−1^ D-xylose as the sole carbon source (see the Section 2).

KH_2_PO_4_ Concentration (g L^−1^)	Volumetric IA Yield (g L^−1^)	Specific Molar IA Yield (Y_p/s_)	Maximal Biomass Concentration (DCW) (g L^−1^)	Fermentation Duration (h)	Overall D-Xylose Utilization Rate (g L^−1^ h^−1^)	Overall IA Production Rate (g L^−1^ h^−1^)
0.04	24.6 ± 2.1	0.57 ± 0.04	4.60 ± 0.8	132	0.38	0.19 ± 0.02
0.10	28.5 ± 2.0	0.66 ± 0.04	5.10 ± 0.8	120	0.42	0.23 ± 0.02
0.80	21.6 ± 1.8	0.50 ± 0.04	7.57 ± 0.6	108	0.46	0.20 ± 0.02

## Data Availability

The original contributions presented in the study are included in the article/Supplementary Materials, further inquiries can be directed to the corresponding author.

## Supplementary Materials

The following supporting information can be downloaded at: https://www.mdpi.com/article/10.3390/jof12010014/s1, Figure S1: Chemical structure of itaconic acid (IA); Table S1: Primers used for qPCR analysis in this study; Table S2: Relevant literature on the relationship between itaconic acid accumulation and initial phosphate concentration in the growth media of *Aspergillus terreus* cultures; Table S3: Itaconic acid production (final volumetric yield; g L^−1^) of *Aspergillus terreus* NRRL 1960 cultures as a function of the initial phosphate ion concentration (g L^−1^) in the growth medium. Mycelia grew in a production-optimized liquid medium initially containing 50 g L^−1^ D-xylose as the sole carbon source in 500-mL shake-flasks (see the Materials and Methods section). While production values numerically differ from those obtained in 6-L scale bioreactors under identical cultivation conditions (see Table 1), these differences are statistically non-significant.
