# Supplementary material for: Extracellular Phosphate Availability Impacts Aspergillus terreus Itaconic Acid Fermentation via Biomass-Specific Product Yield
Source: J Fungi (Basel). 2025 Dec 25;12(1):14. doi: 10.3390/jof12010014 (PMC12843280; doi:10.3390/jof12010014)
Supplement: Supplementary file 1 [file jof-12-00014-s001.zip › jof-4048241-supplementary.pdf]

Supplementary Material (online)

associated with

Extracellular phosphate availability impacts *Aspergillus terreus* itaconic acid fermentation via biomass-specific product yield

Ákos P. Molnár[1], István Bakondi-Kovács[1], Vivien Bíró[1], Alexandra Márton[1], István S. Kolláth[1], Erzsébet Fekete[1*], Norbert Ág[1], Erzsébet Sándor[2], András Csótó[3], Béla Kovács[2], Christian P. Kubicek[4], and Levente Karaffa[1]

[1]Department of Biochemical Engineering, Faculty of Science and Technology, University of Debrecen, Debrecen, Hungary
[2]Institute of Food Science, Faculty of Agricultural and Food Science and Environmental Management, University of Debrecen, Debrecen, Hungary
[3]Institute of Plant Protection, Faculty of Agricultural and Food Science and Environmental Management, University of Debrecen, H-4032 Debrecen, Hungary;
[4]Institute of Chemical, Environmental and Bioscience Engineering, TU Wien, Vienna, Austria
* Correspondence: Erzsébet Fekete, kicsizsoka@yahoo.com

Supplementary Figure S1: Chemical structure of itaconic acid (IA).
Supplementary Table S1: Primers used for qPCR analysis in this study.
Supplementary Table S2: Relevant literature on the relationship between itaconic acid accumulation and initial phosphate concentration in the growth media of *Aspergillus terreus* cultures.
Supplementary Table S3: Itaconic acid production (final volumetric yield; g L^{-1}) of *Aspergillus terreus* NRRL 1960 cultures as a function of the initial phosphate ion concentration (g L^{-1}) in the growth medium. Mycelia grew in a production-optimized liquid medium initially containing 50 g L^{-1} D-xylose as the sole carbon source in 500-mL shake-flasks (see the Materials and Methods section). While production values numerically differ from those obtained in 6-L scale bioreactors under identical cultivation conditions (see Table 1), these differences are statistically non-significant.

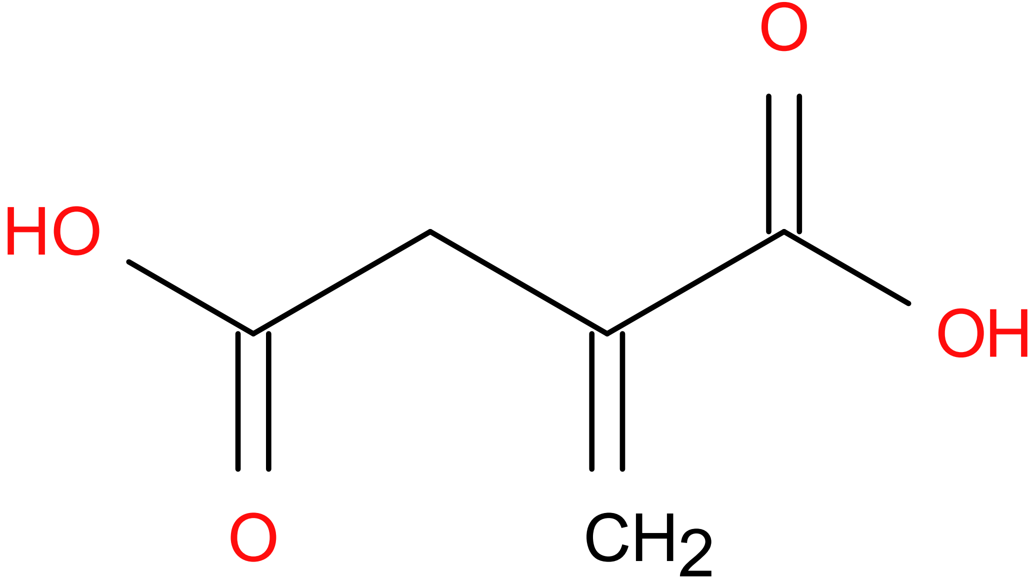

Supplementary Figure S1. Chemical structure of itaconic acid (IA).

Supplementary Table S1. Primers used for qPCR analysis in this study.

Gene to be detected	Forward primer (5'-3')	Reverse primer (5'-3')
ATET_05999 aox1	GACAACGGATGGATCGAAACGC	GCGGCGAAACCAGGTACGAGA
ATET_06973 actA	GAAGTGTGATGTTGATGTCCGT	CGATCCAGACGGAGTATTTACG

Supplementary Table S2. Relevant literature on the relationship between itaconic acid accumulation and initial phosphate concentration in the growth media of *Aspergillus terreus* cultures.

**Reference**	**KH_2PO_4 concentration ($g\ L^{-1}$)**	**Carbon source**	**Initial carbon source concentration ($g\ L^{-1}$)**	**Volumetric IA yield* ($g\ L^{-1}$)**	**Strain (*Aspergillus terreus*)**
Batti and Schweiger 1963	0.14	sucrose	130	≈80	N. A.
Kautola et al., 1985	0.088	D-glucose; D-xylose	55; 67 or 100	30	NRRC 1960
Petruccioli et al., 1999	0.11	D-glucose, starch, starch hydrolysate	60 or 80	19.8	NRRL 1960
Kuenz et al., 2012	0.1	D-glucose	180	86.2	DSM 23081
Hevekerl et al., 2014	0.1; 0.8	D-glucose	180	92,4; 88.5	DSM 23081
Karaffa et al., 2015	0.1	D-glucose	150	≈100	NRRL 1960
Krull et al., 2017	0.1; 0.8	D-glucose	≥180	160	DSM 23081
Kreyenschulte et al., 2018	0.8	D-glucose	180	70	DSM 23081
Saha and Kennedy, 2019	0.008; 0.08; 0.4; 0.8; 1.6	D-glucose	80	50>	NRRL 1972
Saha et al., 2019	0; 0.1; 0.4; 0.8; 1.6; 2.4; 3.2	D-glucose, D-xylose, L-arabinose	80	50>	NRRL 1972
Saha and Kennedy, 2020	0.045; 0.09; 0.135; 0.18; 0.8	D-glucose, D-xylose, L-arabinose	80	50>	NRRL 1972

***Maximal IA concentration reached in cultures**

References

Batti, M.L., and Schweigert, L.B. (1963) Process for the production of itaconic acid. *US Patent 3078217*, 19 February.

Hevekerl, A., Kuenz, A., and Vorlop, K.D. (2014). Filamentous fungi in microtiter plates—an easy way to optimize itaconic acid production with *Aspergillus terreus*. *Applied Microbiology and Biotechnology*, 98(16), 6983-6989. doi: 10.1007/s00253-014-5743-2.

Karaffa, L., Díaz, R., Papp, B., Fekete, E., Sándor, E., and Kubicek, C.P. (2015) A deficiency of manganese ions in the presence of high sugar concentrations is the critical parameter for achieving high yields of itaconic acid by *Aspergillus terreus*. *Applied Microbiology and Biotechnology*, 99, pp. 7937-7944.

Kautola, H., Vahvaselkä, M., Linko, Y.-Y. , and Linko, P. (1985) Itaconic acid production by immobilized *Aspergillus terreus* from xylose and glucose. *Biotechnology Letters*, 7(3), pp. 167-172. doi: 10.1007/BF01027812.

Kreyenschulte, D., Heyman, B., Eggert, A., Maßmann, T., Kalvelage, C., Kossack, R., Regestein, L., Jupke, A., and Büchs, J. (2018) In situ reactive extraction of itaconic acid during fermentation of *Aspergillus terreus*. *Biochemical Engineering Journal*, 135, pp. 133-141. doi: 10.1016/j.bej.2018.04.014.

Krull, S., Hevekerl, A., Kuenz, A., and Prüße, U. (2017) Process development of itaconic acid production by a natural wild-type strain of *Aspergillus terreus* to reach industrially relevant final titers. *Applied Microbiology and Biotechnology*, 101(10), pp. 4063-4072. doi: 10.1007/s00253-017-8192-x.

Kuenz, A., Gallenmüller, Y., Willke, T., and Vorlop, K.D. (2012) Microbial production of itaconic acid: developing a stable platform for high product concentrations. *Applied Microbiology and Biotechnology*, 96(5), pp. 1209-1216.

Petruccioli, M., Pulci, V., and Federici, F. (1999) Itaconic acid production by *Aspergillus terreus* on raw starchy materials. *Letters in Applied Microbiology*, 28(4), pp. 309-312. doi: 10.1046/j.1365-2672.1999.00528.x.

Saha, B.C., and Kennedy, G.J. (2019) Phosphate limitation alleviates the inhibitory effect of manganese on itaconic acid production by *Aspergillus terreus*. *Biocatalysis and Agricultural Biotechnology*, 18, p. 101016. doi: 10.1016/j.bcab.2019.01.054.

Saha, B.C., and Kennedy, G.J. (2020) Efficient itaconic acid production by *Aspergillus terreus*: Overcoming the strong inhibitory effect of manganese. *Biotechnology Progress*, 36(2), p. e2939. doi: 10.1002/btpr.2939.

Saha, B.C., Kennedy, G.J., Bowman, M.J., Qureshi, N., and Dunn, R.O. (2019) Factors affecting production of itaconic acid from mixed sugars by *Aspergillus terreus*. *Applied Biochemistry and Biotechnology*, 187(2), pp. 449-460. doi: 10.1007/s12010-018-2831-2.

Supplementary Table S3. Itaconic acid production (final volumetric yield; g L^{-1}) of *Aspergillus terreus* NRRL 1960 cultures as a function of the initial phosphate ion concentration (g L^{-1}) in the growth medium. Mycelia grew in a production-optimized liquid medium initially containing 50 g L^{-1} D-xylose as the sole carbon source in 500-mL shake-flasks (see the Materials and Methods section). While production values numerically differ from those obtained in 6-L scale bioreactors under identical cultivation conditions (see Table 1), these differences are statistically non-significant.

KH_2PO_4 concentration	0.04	0.06	0.08	0.1	0.2	0.4	0.8	2.0	4.0
Itaconic acid production	23.9 ± 2.0	26.1 ± 1.3	27.9 ± 1.4	28.3 ± 2.1	27.7 ± 1.4	25.9 ± 1.3	24.1 ± 1.5	22.8 ± 1.6	21.4 ± 1.2
